# Supplementary material for: Exposure of mice to environmentally relevant per- and polyfluoroalkyl substances (PFAS) alters the sperm epigenome
Source: Commun Biol. 2025 Oct 28;8:1487. doi: 10.1038/s42003-025-08865-4 (PMC12568938; doi:10.1038/s42003-025-08865-4)
Supplement: Supplementary file 1 — Supplementary Information [file 42003_2025_8865_MOESM1_ESM.pdf]

Supplementary Information

Manuscript Title: Exposure of mice to environmentally relevant per- and polyfluoroalkyl substances (PFAS) alters the sperm epigenome

Authors:

Leah Gillespie[1,2*], Jacinta H. Martin[1,2,†,*], Amanda L. Anderson[1,2], Ilana R. Bernstein[1,2], Simone J. Stanger[1,2], Natalie A. Trigg[1,2,3], John E. Schjenken[1,2], Anne-Louise Gannon[1,2], Shanu Parameswaran[1,2], Shannon P. Smyth[1,2,4], Colin C. Conine[3,5], Reena Desai[6], David J. Handelsman[6], Geoffry N. De Iuliis[1,2], Andrew L. Eamens[7], Matthew D. Dun[8,9], Brett D. Turner[10,11], Shaun D. Roman[12], Mark P. Green[4], Brett Nixon[1,2]

*These authors contributed equally.

Affiliations:

[1] School of Environmental and Life Sciences, University of Newcastle, Callaghan, NSW 2308, Australia.

[2] Infertility and Reproduction Research Program, Hunter Medical Research Institute, New Lambton Heights, Newcastle, NSW 2305, Australia.

[3] Division of Neonatology, Children's Hospital of Philadelphia. Philadelphia, PA, USA.

[4] School of BioSciences, Faculty of Science, University of Melbourne, Victoria 3010, Australia.

[5] Departments of Genetics and Pediatrics - Penn Epigenetics Institute, Institute of Regenerative Medicine, and Center for Reproduction and Women's Health, University of Pennsylvania Perelman School of Medicine. Philadelphia, PA, USA.

[6] ANZAC Research Institute, University of Sydney, Sydney, NSW 2139, Australia.

[7] School of Health, University of the Sunshine Coast, Maroochydore, QLD 4558, Australia.

[8] Cancer Signalling Research Group, School of Biomedical Sciences and Pharmacy, College of Health, Medicine and Wellbeing, University of Newcastle, Callaghan, NSW 2308, Australia.

[9] Precision Medicine Research Program, Hunter Medical Research Institute, New Lambton Heights, Newcastle, NSW 2305, Australia.

[10] Centre for Technology in Water and Wastewater, School of Civil and Environmental Engineering, University of Technology Sydney, Ultimo, NSW 2007, Australia.
[11] Priority Research Centre for Geotechnical Science and Engineering, University of Newcastle, Callaghan, NSW 2308, Australia.
[12] NSW Health Pathology, Newcastle, NSW 2300, Australia.

† Correspondence:
Dr Jacinta Martin
Address: LS-440 Life Sciences: University Drive, Callaghan, NSW 2308, Australia
Phone: +61 2 491 38289
Email: jacinta.martin@newcastle.edu.au

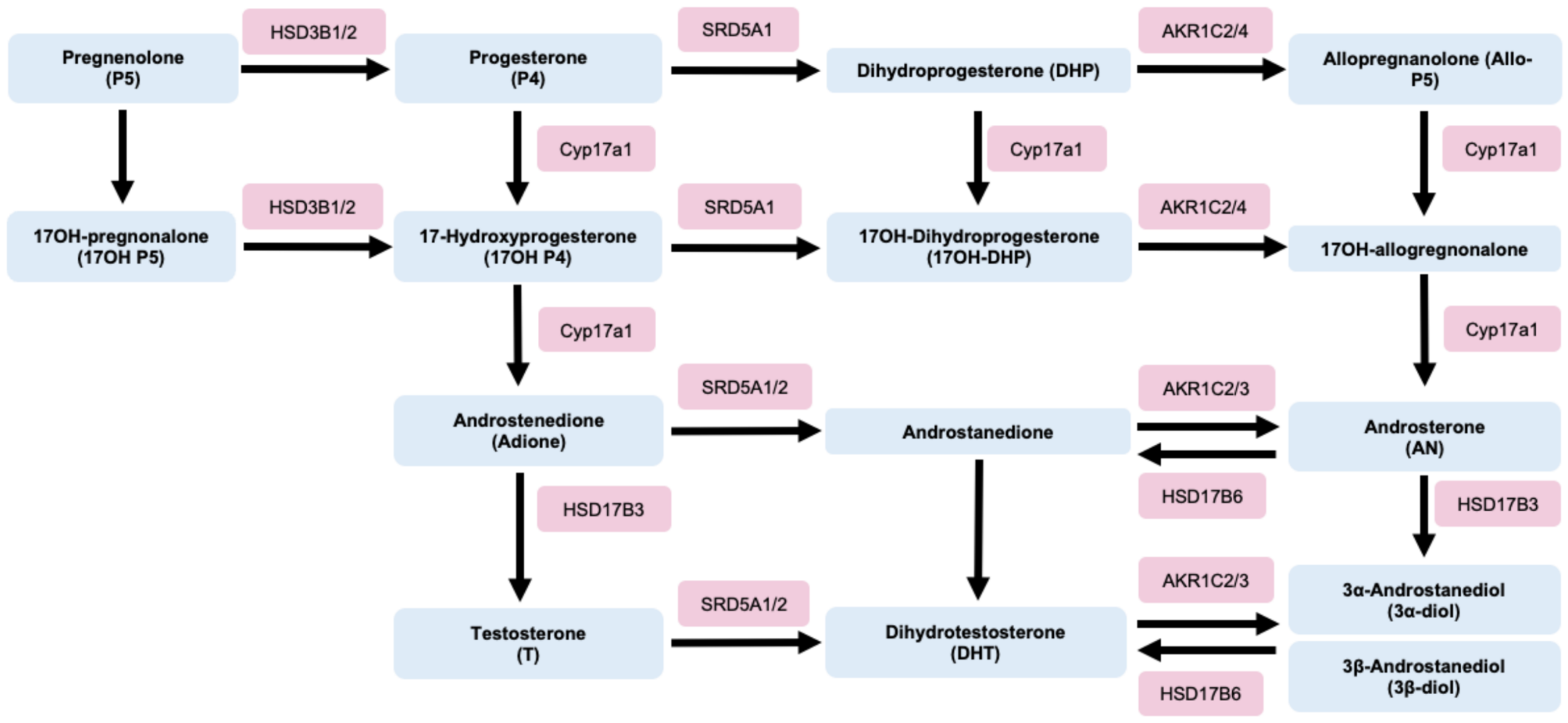

Supplementary Figure 1: Representative schematic of the hormone synthesis pathway with key enzymes (shown in pink) responsible for catalyzing the interconversion of these steroids (shown in blue).

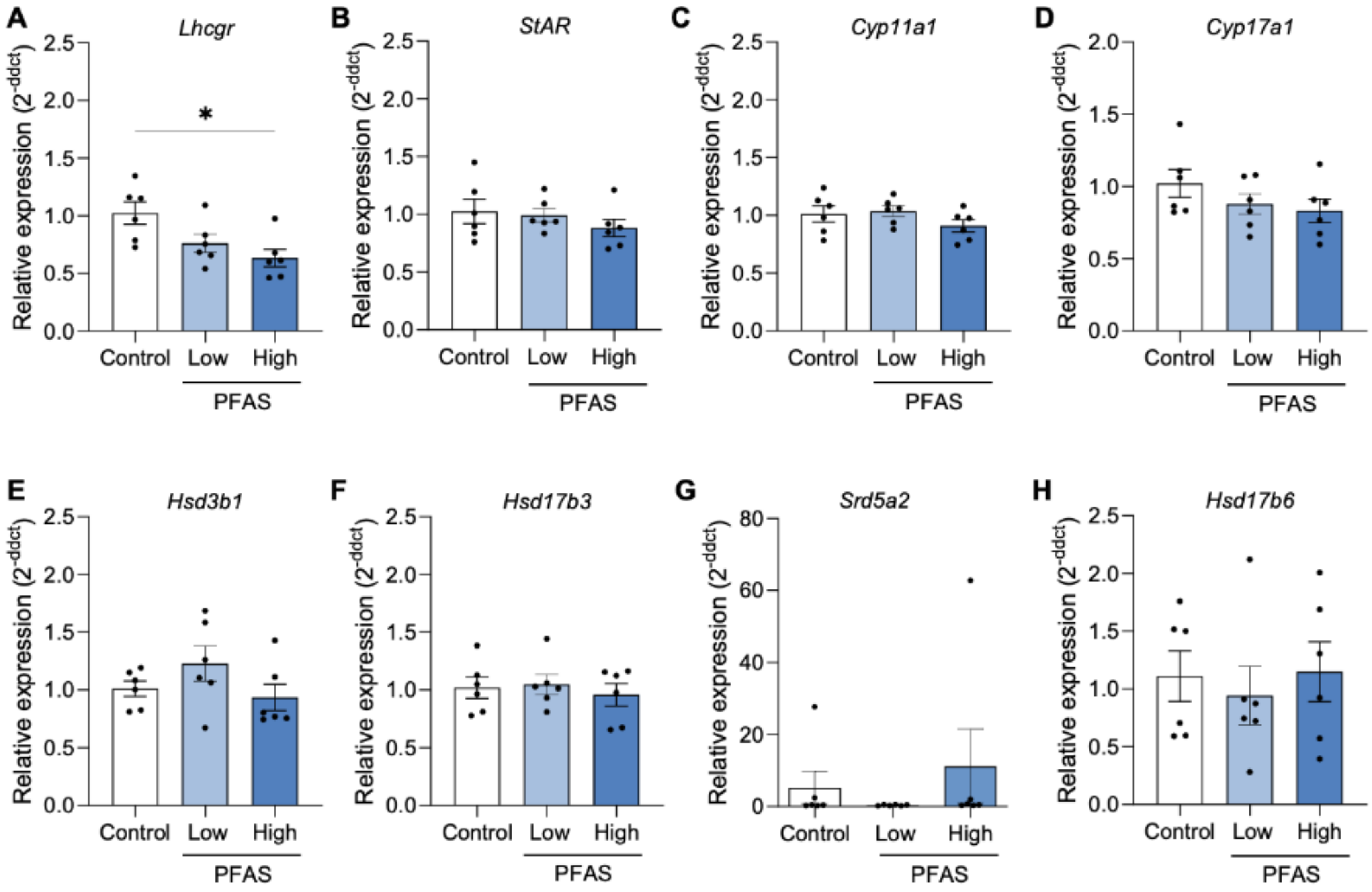
A
Lhcgr
B
StAR
C
Cyp11a1
D
Cyp17a1
E
Hsd3b1
F
Hsd17b3
G
Srd5a2
H
Hsd17b6
Relative expression (2^{-ddct})
Control
Low
High
PFAS
*

Supplementary Figure 2: Quantitative assessment of steroidogenic enzyme gene expression in the testes. Real-time quantitative polymerase chain reaction (RT-qPCR) was employed to assess transcript levels of eight key steroidogenic enzyme transcripts in the testis of per- and polyfluoroalkyl substances (PFAS) exposed mice. Genes assessed include **(A)** luteinizing hormone/choriogonadotropin receptor (*Lhcgr)*, **(B)** steroidogenic acute regulatory protein *(StAR)*, **(C)** cytochrome P450 11A1 (*Cyp11a1)*, **(D)** cytochrome P450 17A1 (*Cyp17a1)*, **(E)** 3beta-hydroxysteroid dehydrogenase/delta(5)-delta(4)isomerase type I (*Hsd3b1)*, **(F)** hydroxysteroid 17-beta dehydrogenase 3 (*Hsd17b3)*, **(G)** steroid 5-alpha-reductase 2 (*Srd5a2)*, **(H)** hydroxysteroid 17-beta dehydrogenase 6 (*Hsd17b6)*. Each gene was normalized to beta actin using the delta delta CT method ($^{2\text{-ddct}}$) and presented as mean ± SEM, calculated on the basis of n=6 biological replicates per exposure group. Data were subjected to one-way ANOVA with Tukey's multiple comparisons test. * $p < 0.05$.

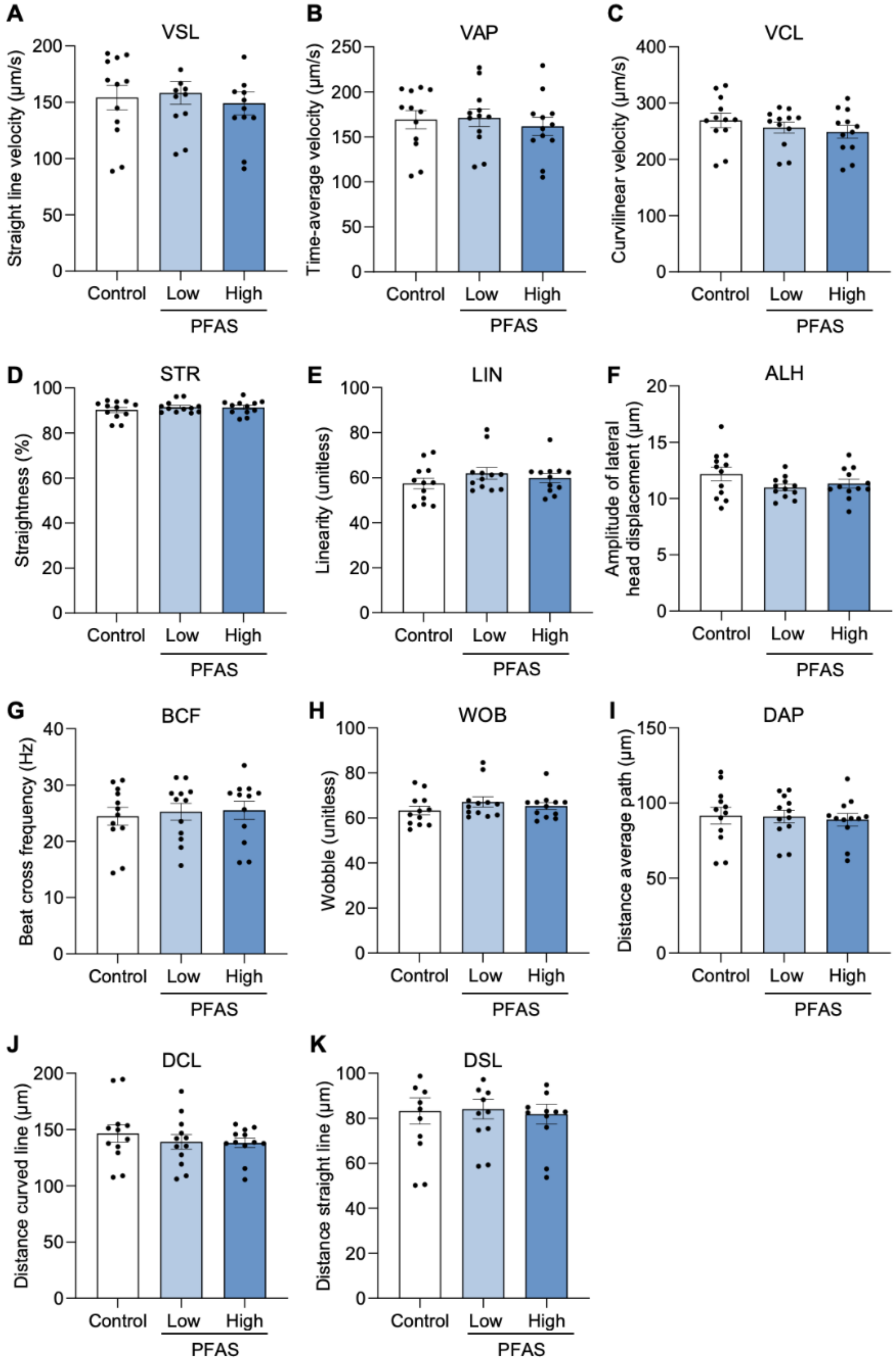

A
VSL
Straight line velocity (µm/s)
B
VAP
Time-average velocity (µm/s)
C
VCL
Curvilinear velocity (µm/s)
D
STR
Straightness (%)
E
LIN
Linearity (unitless)
F
ALH
Amplitude of lateral head displacement (µm)
G
BCF
Beat cross frequency (Hz)
H
WOB
Wobble (unitless)
I
DAP
Distance average path (µm)
J
DCL
Distance curved line (µm)
K
DSL
Distance straight line (µm)
Control
Low
High
PFAS

Supplementary Figure 3: Computer Assisted Sperm Analysis assessment of the effect of per- and polyfluoroalkyl substances (PFAS) exposure on sperm motility parameters. Cauda epididymal spermatozoa isolated from control (unexposed) or PFAS-exposed mice were assessed for multiple motility parameters using computer assisted sperm analysis (CASA). Parameters included **(A)** straight line velocity (VSL), **(B)** the time-average velocity (VAP), **(C)** curvilinear velocity (VCL), **(D)** straightness (STR), **(E)** linearity (LIN), **(F)** amplitude of lateral head displacement (ALH), **(G)** beat cross frequency (BCF), **(H)** wobble (WOB), **(I)** distance average path (DAP), **(J)** distance curved line (DCL) and **(K)** distance straight line (DSL). Data are presented as mean ± SEM, calculated on the basis of n=12 biological replicates per exposure group. Data were subjected to one-way ANOVA with Tukey's multiple comparisons test.

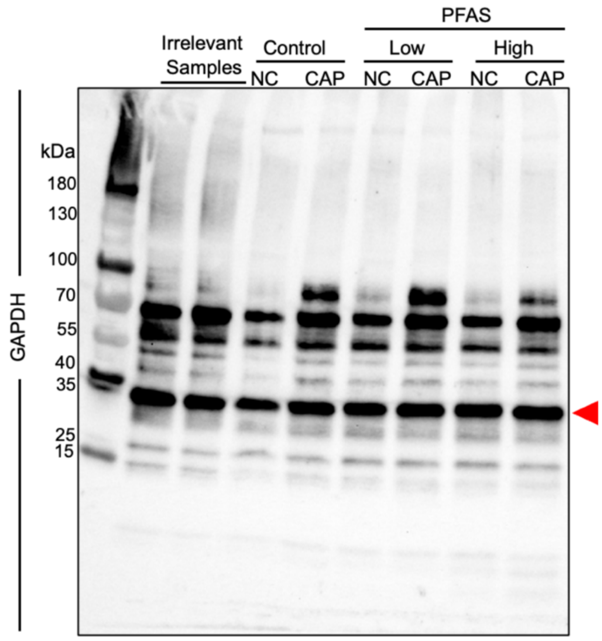

Supplementary Figure 4: Full-length western blot corresponding to the glyceraldehyde-3-phosphate dehydrogenase (GAPDH) loading control shown in Figure 6. This image presents the complete, uncropped blot, with molecular weight markers (kDa) indicated on the left. The red arrow denotes the GAPDH band. Additional banding present on the membrane represents residual signal from the prior phosphotyrosine staining. Lanes 1 and 2 contain samples from an unrelated project, run on the same blot to conserve reagents and supplies, and are not part of the analysis reported here.

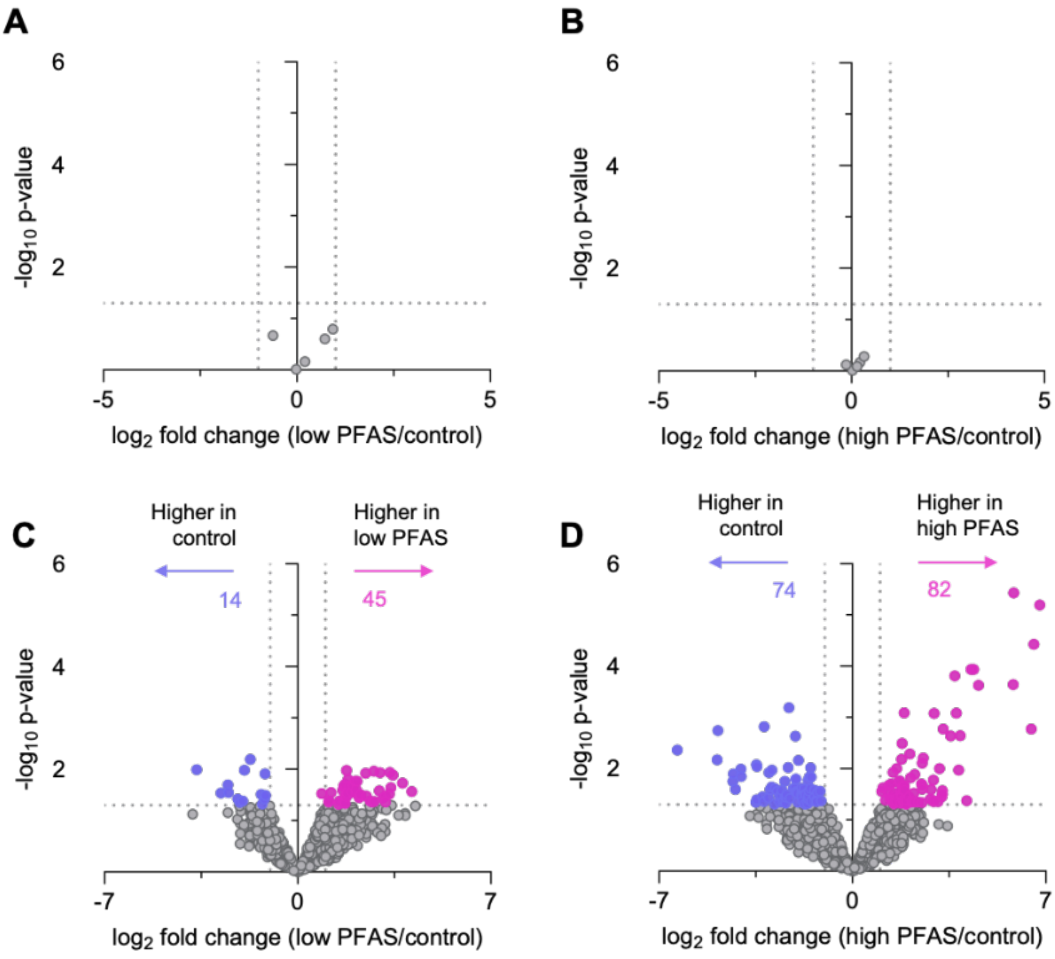

Supplementary Figure 5: The impact of per- and polyfluoroalkyl substances (PFAS) exposure on the sperm small non-coding RNA profile. Volcano plots illustrating the $\log_2$ fold change and $\log_{10}$ *p*-value for **(A-B)** rRNA fragments and **(C-D)** mRNA fragments identified in the spermatozoa of males exposed to low PFAS (left) and high PFAS (right). Colored dots are used to indicate differentially accumulated sncRNAs as determined by DESeq2. Purple is used to represent reduced abundance in PFAS exposed spermatozoa and pink indicates an increased abundance. The applied cutoff criteria for altered sncRNAs was a fold-change ≥ 1.5 and *p*-value ≤ 0.05. All analyses were based on n=3-4 male mice per exposure group.

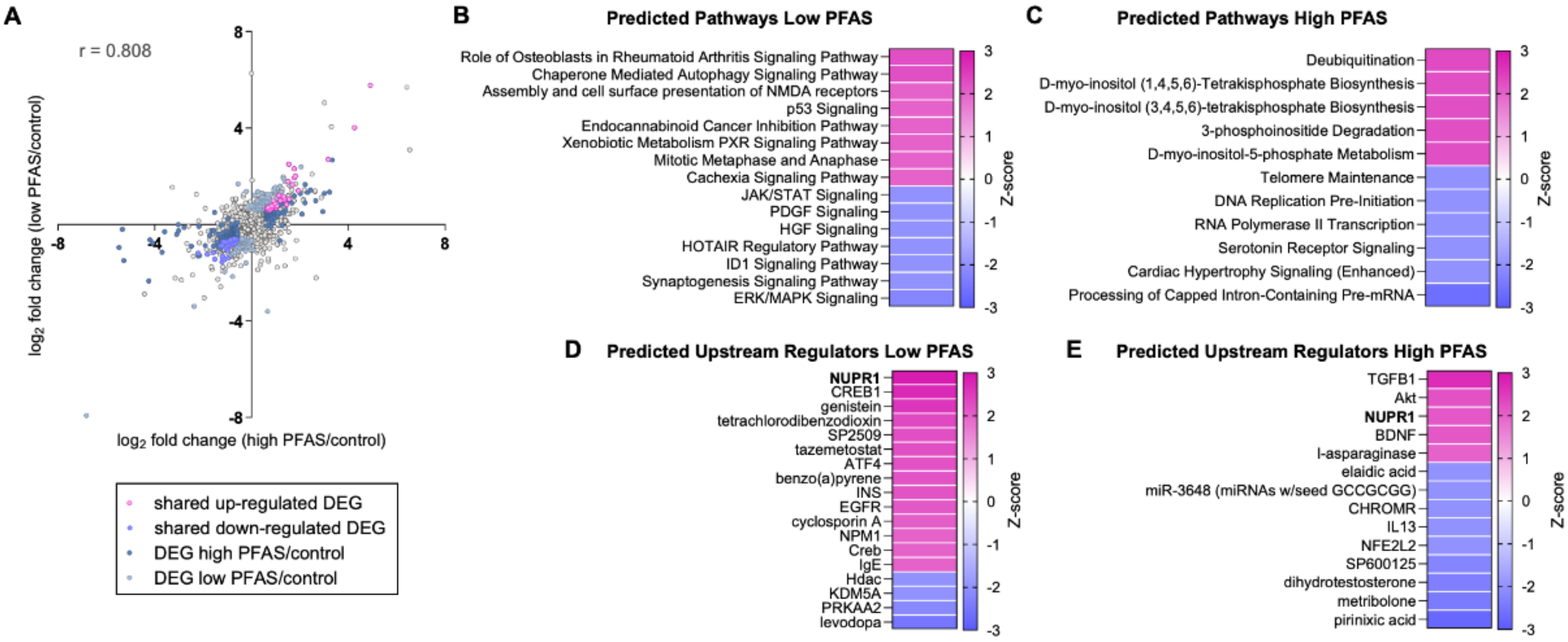
A
r = 0.808
log2 fold change (low PFAS/control)
log2 fold change (high PFAS/control)
shared up-regulated DEG
shared down-regulated DEG
DEG high PFAS/control
DEG low PFAS/control
B
Predicted Pathways Low PFAS
Role of Osteoblasts in Rheumatoid Arthritis Signaling Pathway
Chaperone Mediated Autophagy Signaling Pathway
Assembly and cell surface presentation of NMDA receptors
p53 Signaling
Endocannabinoid Cancer Inhibition Pathway
Xenobiotic Metabolism PXR Signaling Pathway
Mitotic Metaphase and Anaphase
Cachexia Signaling Pathway
JAK/STAT Signaling
PDGF Signaling
HGF Signaling
HOTAIR Regulatory Pathway
ID1 Signaling Pathway
Synaptogenesis Signaling Pathway
ERK/MAPK Signaling
Z-score
C
Predicted Pathways High PFAS
Deubiquitination
D-myo-inositol (1,4,5,6)-Tetrakisphosphate Biosynthesis
D-myo-inositol (3,4,5,6)-tetrakisphosphate Biosynthesis
3-phosphoinositide Degradation
D-myo-inositol-5-phosphate Metabolism
Telomere Maintenance
DNA Replication Pre-Initiation
RNA Polymerase II Transcription
Serotonin Receptor Signaling
Cardiac Hypertrophy Signaling (Enhanced)
Processing of Capped Intron-Containing Pre-mRNA
Z-score
D
Predicted Upstream Regulators Low PFAS
NUPR1
CREB1
genistein
tetrachlorodibenzodioxin
SP2509
tazemetostat
ATF4
benzo(a)pyrene
INS
EGFR
cyclosporin A
NPM1
Creb
IgE
Hdac
KDM5A
PRKAA2
levodopa
Z-score
E
Predicted Upstream Regulators High PFAS
TGFB1
Akt
NUPR1
BDNF
l-asparaginase
elaidic acid
miR-3648 (miRNAs w/seed GCCGCGG)
CHROMR
IL13
NFE2L2
SP600125
dihydrotestosterone
metribolone
pirinixic acid
Z-score

Supplementary Figure 6: Canonical pathways and upstream regulators predicted to be affected by altered embryonic gene expression. (A) Scatter plot comparing the log2 fold-change of gene expression in embryos fertilized by the spermatozoa of high per- and polyfluoroalkyl substances (PFAS) males compared to control (x-axis) versus that of equivalent embryos fertilized by the spermatozoa of low PFAS males compared to control (y-axis). To differentiate differentially expressed genes (DEGs), green dots indicate shared (across both exposure groups) while up- and down-regulated DEGs are represented as pink and purple dots respectively. Light and dark blue dots highlight DEGs in the embryos fertilized by sperm from the low and high PFAS males, respectively. Ingenuity pathway analysis (IPA) software was used to identify the top-ranking ($p \leq 0.05$; Z-score ≥ 2 or ≤ -2) canonical pathways in both the **(B)** low and **(C)** high PFAS groups, as well as upstream regulators in the **(D)** low and **(E)** high PFAS groups predicted to be affected by embryonic DEGs ($p \leq 0.05$; Z-score ≥ 2 or ≤ -2. Conserved upstream regulators predicted in both low and high PFAS are shown in bold.

Supplementary Table 1. Primer sequences used for real-time quantitative polymerase chain reaction. This table contains the sequences for the forward and reverse primers used for the assessment of steroidogenic enzyme gene expression in the testes.

Gene Name	Gene symbol	Forward Primer	Reverse Primer
Beta-actin	*β actin*	TGTGATGGTGGGTATGGGTC	ACACGCAGCTCATTGTA
Luteinizing hormone/choriogonadotropin receptor	*Lhcgr*	CCTGGAAGGTGCCACTGT	GGGACGACGCTAATCTCG
Steroidogenic acute regulatory protein	*StAR*	CCAGGAAGGCTGGAAGAAGG	CCCACATCTGGCACCATCTT
Cytochrome P450 11A1	*Cyp11a1*	TGATGCCTGAGAACCCCATC	TCTTTTCTGGTCACGGCTGG
Cytochrome P450 17A1	*Cyp17a1*	CATCCCACACAAGGCTAACA	CAGTGCCCAGAGATTGATGA
3β-hydroxysteroid dehydrogenase	*Hsd3b1*	GAACTGCAGGAGGTCAGAGC	GCACTGGGCATCCAGAAT
17β-hydroxysteroid dehydrogenase 3	*Hsd17b3*	AATATGTCACGATCGGAGCTG	GAAGGGATCCGGTTCAGAAT
Steroid 5 alpha-reductase 2	*Srd5a2*	CGCTCCCTCTTTGGACCT	CCTCTGGTGAGCAATGAGTAAA
17β-hydroxysteroid dehydrogenase 6	*Hsd17b6*	CGAGGAGCTGAGGAACAAGA	CCCAGAGTCCTCTGTCTCCA

Supplementary Table 2. Summary of proportional changes in the small non-coding RNA (sncRNA) composition of spermatozoa isolated from mice exposed to per- and polyfluoroalkyl substances (PFAS). Upon dissection, mature caudal spermatozoa were isolated and prepared for sncRNA isolation and sequencing. [1]Data are presented as the average percentage of total sncRNA of each subclass based on n=3-4 biological replicates per exposure group. Data were subjected to two-way ANOVA with Tukey's multiple comparisons test. [2]NS = not statistically significant. miRNA = microRNA, tRFs/tDRs/tsRNA = tRNA fragments, piRNA = piwi-interacting RNAs, mRNA = messenger RNA, rRNA = ribosomal RNA.

sncRNA Subclass	Control (%)[1]	Low PFAS-exposed (%)[1]	High PFAS-exposed (%)[1]	Statistical significance[2]
miRNA	19.55	20.19	24.85	NS
tRFs	39.84	27.32	27.41	NS
piRNA	10.77	11.88	11.34	NS
mRNA	6.22	13.37	5.28	NS
rRNA	23.28	26.68	30.76	NS
