# Supplementary material for: Exposure of mice to environmentally relevant per- and polyfluoroalkyl substances (PFAS) alters the sperm epigenome
Source: Commun Biol. 2025 Oct 28;8:1487. doi: 10.1038/s42003-025-08865-4 (PMC12568938; doi:10.1038/s42003-025-08865-4)
Supplement: Supplementary file 2 — Description of Additional Supplementary Materials [file 42003_2025_8865_MOESM2_ESM.pdf]

Description of Additional Supplementary Files

File name: Supplementary Data 1

Description: Comparative analysis of small non-coding RNA (sncRNA) abundance profiles derived from spermatozoa from either low per- and polyfluoroalkyl substances (PFAS) exposed or control (unexposed) male mice. sncRNA was isolated from exposed mice following the exposure regimen. Comparative analysis was performed using DESeq2 package in R Statistical Software to determine differential abundance of sncRNA in low PFAS and control cauda spermatozoa. sncRNAs were defined as differentially expressed when they met the criteria of a fold-change ≥ 2 or ≤ -2 and a p-value ≤ 0.05. 1sncRNAs highlighted in blue indicate differentially accumulated sncRNAs. 2baseMean = the average of the normalized count values, dividing by size factors, of all samples in the data frame.

File name: Supplementary Data 2

Description: Comparative analysis of small non-coding RNA (sncRNA) abundance profiles derived from sperm from either high per- and polyfluoroalkyl substances (PFAS) exposed or control (unexposed) male mice. sncRNA was isolated from exposed mice following the exposure regimen. Comparative analysis was performed using DESeq2 package in R Statistical Software to determine differential abundance of sncRNA in high PFAS and control cauda spermatozoa. sncRNAs were defined as differentially expressed when they met the criteria of a fold-change ≥ 2 or ≤ -2 and a p-value ≤ 0.05. 1sncRNAs highlighted in blue indicate differentially accumulated sncRNAs. 2baseMean = the average of the normalized count values, dividing by size factors, of all samples in the data frame.

File name: Supplementary Data 3

Description: Comparative analysis of 4-cell embryo gene expression profiles generated following in vitro fertilization (IVF) using sperm from males exposed to the low per- and polyfluoroalkyl substances (PFAS) cocktail.

The gene expression profiles of 4-cell embryos generated from low PFAS-exposed, or control spermatozoa were assessed. Raw read counts were imported into R statistical software for comparative analysis using DESeq2. Genes with an average transcript per million of ≥ 5 in one of the groups were retained for analysis, identifying 7,417 assessable genes. Genes were defined as differentially expressed (DEGs) when they met the criteria of a fold-change ≥ 1.5 or ≤ -1.5 and a p-value ≤ 0.05. 1Genes highlighted in blue represent the 67 DEGs altered in both low- and high-PFAS embryos. 2baseMean = the average of the normalized count values, dividing by size factors, of all samples in the data frame.

File name: Supplementary Data 4

Description: Comparative analysis of 4-cell embryo gene expression profiles generated following in vitro fertilization (IVF) using spermatozoa from mice exposed to the high per- and polyfluoroalkyl substances (PFAS) cocktail.

The gene expression profiles of 4-cell embryos generated from high PFAS-exposed, or control spermatozoa were assessed. Raw read counts were imported into R statistical

software for comparative analysis using DESeq2. Genes with an average transcript per million of ≥ 5 in one of the groups were retained for analysis, identifying 7,417 assessable genes. Genes were defined as differentially expressed (DEGs) when they met the criteria of a fold-change ≥ 1.5 or ≤ -1.5 and a p-value ≤ 0.05. 1Genes highlighted in blue represent the 67 DEGs altered in both low- and high-PFAS embryos. 2baseMean = the average of the normalized count values, dividing by size factors, of all samples in the data frame.

File name: Supplementary Data 5

Description: Source data
