# Supplementary material for: Exposure of mice to environmentally relevant per- and polyfluoroalkyl substances (PFAS) alters the sperm epigenome
Source: Commun Biol. 2025 Oct 28;8:1487. doi: 10.1038/s42003-025-08865-4 (PMC12568938; doi:10.1038/s42003-025-08865-4)
Supplement: Supplementary file 8 — Reporting Summary [file 42003_2025_8865_MOESM8_ESM.pdf]

nature portfolio

Corresponding author(s): Jacinta Martin

Last updated by author(s): Sep 5, 2025

Reporting Summary

Nature Portfolio wishes to improve the reproducibility of the work that we publish. This form provides structure for consistency and transparency in reporting. For further information on Nature Portfolio policies, see our Editorial Policies and the Editorial Policy Checklist.

Statistics

For all statistical analyses, confirm that the following items are present in the figure legend, table legend, main text, or Methods section.

n/a	Confirmed	
☐	☒	The exact sample size (*n*) for each experimental group/condition, given as a discrete number and unit of measurement
☐	☒	A statement on whether measurements were taken from distinct samples or whether the same sample was measured repeatedly
☐	☒	The statistical test(s) used AND whether they are one- or two-sided<br>*Only common tests should be described solely by name; describe more complex techniques in the Methods section.*
☒	☐	A description of all covariates tested
☐	☒	A description of any assumptions or corrections, such as tests of normality and adjustment for multiple comparisons
☐	☒	A full description of the statistical parameters including central tendency (e.g. means) or other basic estimates (e.g. regression coefficient) AND variation (e.g. standard deviation) or associated estimates of uncertainty (e.g. confidence intervals)
☒	☐	For null hypothesis testing, the test statistic (e.g. *F*, *t*, *r*) with confidence intervals, effect sizes, degrees of freedom and *P* value noted<br>*Give P values as exact values whenever suitable.*
☒	☐	For Bayesian analysis, information on the choice of priors and Markov chain Monte Carlo settings
☒	☐	For hierarchical and complex designs, identification of the appropriate level for tests and full reporting of outcomes
☒	☐	Estimates of effect sizes (e.g. Cohen's *d*, Pearson's *r*), indicating how they were calculated

Our web collection on statistics for biologists contains articles on many of the points above.

Software and code

Policy information about availability of computer code

Data collection

RNA-seq data was collected using NextSeq1000 (Illumina).

Data analysis

For statistical analysis GraphPad Prism version 9 was used.
ImageJ was used to perform densitometry analysis on immunoblots.
Ingenuity Pathway Analysis (Qiagen) was used to interrogate gene pathways.
RNA-seq data were mapped against the Mus musculus genome (mm10) using RSEM and normalized to transcripts per million.
Small RNA-seq data read quality was assessed using FastQC, and adapter sequences were trimmed using trimmomatic. Trimmed reads were mapped sequentially to rRNA mapping reads, miRbase, murine tRNAs, pachytene piRNA clusters , repeatmasker and Refseq using Bowtie 2 and totaled using Feature counts on Via Foundry (formerly DolphinNext) bioinformatics platform (v.1.6.4). To assess the DEGs, data was loaded into R Statistical Software and analyzed using the DESeq2 package.

For manuscripts utilizing custom algorithms or software that are central to the research but not yet described in published literature, software must be made available to editors and reviewers. We strongly encourage code deposition in a community repository (e.g. GitHub). See the Nature Portfolio guidelines for submitting code & software for further information.

Data

Policy information about availability of data

All manuscripts must include a data availability statement. This statement should provide the following information, where applicable:

- Accession codes, unique identifiers, or web links for publicly available datasets
- A description of any restrictions on data availability
- For clinical datasets or third party data, please ensure that the statement adheres to our policy

The gene expression datasets generated and analyzed during the current study are available in the Gene Expression Omnibus repository with accession number GSE271479

Research involving human participants, their data, or biological material

Policy information about studies with human participants or human data. See also policy information about sex, gender (identity/presentation), and sexual orientation and race, ethnicity and racism.

Reporting on sex and gender	This study does not include and research on human participants, their data or biological material.
Reporting on race, ethnicity, or other socially relevant groupings	This study does not include and research on human participants, their data or biological material.
Population characteristics	This study does not include and research on human participants, their data or biological material.
Recruitment	This study does not include and research on human participants, their data or biological material.
Ethics oversight	This study does not include and research on human participants, their data or biological material.

Note that full information on the approval of the study protocol must also be provided in the manuscript.

Field-specific reporting

Please select the one below that is the best fit for your research. If you are not sure, read the appropriate sections before making your selection.

☒ Life sciences ☐ Behavioural & social sciences ☐ Ecological, evolutionary & environmental sciences

For a reference copy of the document with all sections, see nature.com/documents/nr-reporting-summary-flat.pdf

Life sciences study design

All studies must disclose on these points even when the disclosure is negative.

Sample size	For all experiments n =3 was used as the minimum sample size. The only exception is for PFAS measurement and hormone measurement in which not all returned values above the limit of detection. For embryo RNA seq n=3-4 replicates with 8-10 embryos/replicate were used. The sample size of all experiments is clearly outlined in figure legends and/or the methods section.
Data exclusions	Data exclusions were only performed for sequencing experiments using pre-established criteria. Here 1 control embryo sample was removed due to poor read coverage (i.e.had fewer than 9,000 detected transcripts). This likely occurred as a result of either uneven library loading or error in library preparation.
Replication	All experiments were repeated a minimum of 3 times to ensure reproducibility and all attempts at replication were successful. In those exceptions listed above in 'sample size' 6-8 individual biological replicates were initially sent for testing and only those above the limit of detection of the instrument were reported.
Randomization	Randomisation was performed at the point of animal arrival by allocating individuals sequentially into control and treatment groups in an alternating manner to ensure balanced group sizes. This approach ensured an unbiased distribution of animals across groups while maintaining practical feasibility during the allocation process
Blinding	Researchers were not blinded to experimental groups.

Reporting for specific materials, systems and methods

We require information from authors about some types of materials, experimental systems and methods used in many studies. Here, indicate whether each material, system or method listed is relevant to your study. If you are not sure if a list item applies to your research, read the appropriate section before selecting a response.

Materials & experimental systems

n/a	Involved in the study	
☐	☒	Antibodies
☒	☐	Eukaryotic cell lines
☒	☐	Palaeontology and archaeology
☐	☒	Animals and other organisms
☒	☐	Clinical data
☒	☐	Dual use research of concern
☒	☐	Plants

Methods

n/a	Involved in the study	
☒	☐	ChIP-seq
☒	☐	Flow cytometry
☒	☐	MRI-based neuroimaging

Antibodies

Antibodies used	ApopTag Fluorescein in Situ Apoptosis Detection Kit (cat # S7110; Merck).<br>Anti-phosphotyrosine antibodies (PT66; cat # P5872; Merck)
Validation	All antibodies have been validated by vendors and/or previous publications. Details on validation are provided on product details page for commercially available antibodies.

Animals and other research organisms

Policy information about studies involving animals; ARRIVE guidelines recommended for reporting animal research, and Sex and Gender in Research

Laboratory animals	Adult (4-5 week-old) male and female (4-6 week-old) Swiss CD1 mice (SwissTacAusb, Swiss Webster Outbred), derived from the Taconic Swiss Webster lineage, were obtained from Australian BioResources (ABR; Moss Vale, NSW, Australia), or the University of Newcastle Animal Services Unit (NSW, Australia).
Wild animals	This study did not involve wild animals
Reporting on sex	This study focused on sperm biology and therefore we only used male mice for sample collection and female mice as egg donors to assess male fertility and generate embryos.
Field-collected samples	This study did not include field collected samples
Ethics oversight	We have complied with all relevant ethical regulations for animal use.<br>These mice were monitored, handled, and euthanized in accordance with the NSW Animal Research Act 1998, NSW Animal Research Regulation 2010, and the Australian Code for the Care and Use of Animals for Scientific Purposes 8th Edition, and with the ethical approval of the University of Newcastle Animal Care and Ethics Committee (A-2020-009).

Note that full information on the approval of the study protocol must also be provided in the manuscript.

Plants

Seed stocks	This study did not involve plants
Novel plant genotypes	This study did not involve plants
Authentication	This study did not involve plants
